# Transcriptome Analysis of the Regulatory Mechanism of FoxO on Wing Dimorphism in the Brown Planthopper, *Nilaparvata lugens* (Hemiptera: Delphacidae)

**DOI:** 10.3390/insects12050413

**Published:** 2021-05-04

**Authors:** Nan Xu, Sheng-Fei Wei, Hai-Jun Xu

**Affiliations:** 1 State Key Laboratory of Rice Biology, Zhejiang University, Hangzhou 310058, China; 11816017@zju.edu.cn (N.X.); 21816099@zju.edu.cn (S.-F.W.); 2 Ministry of Agriculture Key Laboratory of Molecular Biology of Crop Pathogens and Insect Pests, Zhejiang University, Hangzhou 310058, China; 3 Institute of Insect Sciences, Zhejiang University, Hangzhou 310058, China

**Keywords:** planthopper, wing polyphenism, RNA-seq, FoxO, cell proliferation

## Abstract

**Simple Summary:**

The brown planthopper (BPH) *Nilaparvata lugens* can develop into either long-winged or short-winged adults depending on environmental stimuli received during larval stages. The transcription factor *Nl*FoxO serves as a key regulator determining alternative wing morphs in BPH, but the underlying molecular mechanism is largely unknown. Here, we investigated the transcriptomic profile of forewing and hindwing buds across the 5th-instar stage, the wing-morph decision stage. Our results indicated that *Nl*FoxO modulated the developmental plasticity of wing buds mainly by regulating the expression of cell proliferation-associated genes.

**Abstract:**

The brown planthopper (BPH), *Nilaparvata lugens*, can develop into either short-winged (SW) or long-winged (LW) adults according to environmental conditions, and has long served as a model organism for exploring the mechanisms of wing polyphenism in insects. The transcription factor *Nl*FoxO acts as a master regulator that directs the development of either SW or LW morphs, but the underlying molecular mechanism is largely unknown. Here, we microinjected SW-destined morphs with double stranded-RNA (dsRNA) targeting *NlFoxO* (ds*NlFoxO*) to change them into LW-winged morphs. In parallel, SW-destined morphs microinjected with dsRNA targeting the gene encoding green fluorescence protein (ds*Gfp*) served as a negative control. The forewing and hindwing buds of 5th-instar nymphs collected at 24, 36, and 48 h after eclosion (hAE) were used for RNA sequencing. We obtained a minimum of 43.4 million clean reads from forewing and hindwing buds at a single developmental time. Differentially expressed genes (DEGs) were significantly enriched in various Gene Ontology (GO) terms, including cellular process, binding, and cell part. Kyoto Encyclopedia of Genes and Genomes (KEGG) enrichment pathway analysis showed that up-regulated genes in ds*NlFoxO*-treated forewing and hindwing buds were largely associated with the cell cycle and DNA replication. Furthermore, most up-regulated genes displayed higher expression at 24-, and 36-hAE relative to 48 hAE, indicating that wing cells in LW-destined wings might actively proliferate during the first 36 h in 5th-instar nymphs. Our findings indicated that LW development in BPH was likely dependent on the duration of cell proliferation in the 5th-instar stage, which sheds light on the molecular basis of wing polymorphism in insects.

## 1. Introduction

The brown planthopper (BPH), *Nilaparvata lugens* (Hemiptera: Delphacidae), is the most destructive rice pest in Asia [1]. BPH feeds exclusively on the phloem sap of rice plants and transmits plant viruses such as rice ragged stunt viruses and rice grassy stunt viruses, leading to huge losses in rice yields [2,3]. BPH is one of the most extensively studied wing-dimorphic insects due to the natural occurrence of short- and long-winged (SW and LW) adults [4]. As a hemimetabolous insect, newly hatched first-instar nymphs look like miniature adults, and wing buds grow gradually as the developmental stages advance. Our recent studies showed that the *N. lugens FoxO* homolog *Nl*FoxO relied on the insulin/insulin-like signaling (IIS) activity to direct wing buds developing into SW or LW morphs during the wing-morph decisive stage (the 5th-instar stage). High IIS activity inhibited *Nl*FoxO activity, leading to LW morphs, and vice versa [5,6,7]. However, the molecular basis by which *Nl*FoxO influences alternative wing morphs remains largely unknown.

FoxO transcription factors belong to the large Forkhead family of proteins, which are characterized by a conserved DNA-binding domain termed the “forkhead box” [8]. FoxO proteins are tightly involved in various cellular processes including regulating the expression of genes associated with cell cycle, DNA repair, apoptosis, and energy balance. Loss of *FoxO* is associated with increased cancer in mammals [9,10]. In the nematode worm *Caenorhabditis elegans*, the *FoxO* homolog *daf-16* was initially isolated as a dauer defective mutant [11], and overexpression of *daf-16* can extend lifespan via dauer formation [12]. In addition, FoxO mediates the effects of the IIS pathway in stress resistance, fat storage, immunity, and reproduction [13,14,15,16,17,18]. In the absence of IIS activity, FoxO induces cell cycle arrest and entrance into cellular quiescence [19]. FoxO transcription factors play a major role in G1 arrest by both up-regulating cell cycle inhibitors (p21 and p27) and by repressing cell cycle activators (cyclin D1/D2) [20,21]. In the model insect *Drosophila melanogaster*, fly *FoxO* homolog *dFoxO* mutants were viable and of normal size, but lost protection against oxidative stress [22]. In contrast, ectopic expression of *dFoxO* caused a decrease in cell number and cell size [22,23,24], leading to a reduction in body size.

The emergence of next-generation sequencing technology has profoundly improved our understanding of the molecular basis of wing polymorphism in insects. Our early RNA sequencing (RNA-seq) study identified hundreds of genes including those correlated to respiration and energy metabolism were up-regulated in LW versus SW BPH adults, indicating LW BPH adults might require more energy than SW for flight [25]. In the cotton aphids (*Aphis gossypii*), genes associated with flight-reproduction trade-offs were differentially expressed in winged versus wingless morphs through RNA-seq analysis [26]. In the brown citrus aphid (*Toxoptera citricida*), RNA-seq identified both lipid and glycogen metabolism-associated genes that were differentially expressed between winged and wingless adults, indicating that these genes might contribute to energy metabolism during aphid wing development [27].

In the present study, we microinjected SW-destined morphs with double stranded-RNA (dsRNA) targeting *NlFoxO* (ds*NlFoxO*) to change them into LW-winged morphs. In parallel, SW-destined morphs microinjected with dsRNA targeting the gene encoding green fluorescence protein (ds*Gfp*) served as a negative control. Forwing and hindwing buds were dissected from ds*NlFoxO*- and ds*Gfp*-treated nymphs at the wing-morph decisive stage (the 5th-instar stage), and were then used for RNA-sequencing. Comparative transcriptomic analysis indicated that *Nl*FoxO-regulated alternative wing morphs were likely through modulating genes involved in cell proliferation. These results advanced our understanding of the molecular basis of wing dimorphism in insects.

## 2. Materials and Methods

### 2.1. Insect Colony

The SW *N. lugens* strain (SW > 95%) was initially collected from a rice field in Hangzhou, China, in 2009 [25]. Insects were maintained on rice seedings (rice variety: Xiushui134) in a walk-in chamber at 26 ± 0.5 °C with a relative humidity of 50 ± 10% and a photoperiod of 16:8 h (light: dark).

### 2.2. RNA Extraction and cDNA Library Construction

4th-instar nymphs were collected for microinjection with ds*NlFoxO* to generate LW-destined morphs. For a negative control, 4th-instar nymphs were microinjected with ds*Gfp*, which produced SW-destined adults as the SW *N. lugens* strain. For transcriptome sequencing, forewing and hindwing buds were dissected from 5th-instar nymphs (*n* = 200 for each biological replicate) at 24, 36, and 48 h after ecdysis (hAE). Three independent biological replicates were performed at each designed time. Total RNA was isolated from wing buds using TRIzol (Takara, Dalian, China), and the RNA concentration and quality were determined using a NanoDrop ND-2000 spectrophotometer (Thermo Fisher, Waltham, MA, USA). RNA samples (>2 μg) were subsequently used for cDNA library construction using a NEBNext Ultra RNA Library Prep Kit for Illumina (NEB, Ipswich, MA, USA) according to manufacturer’s recommendations, and index codes were added to each sequence. Library fragments of 250–300 bp in length were preferentially purified using an AMPure XP system (Beckman Coulter, Woerden, The Netherlands), and library quality was assessed using the Agilent Bioanalyzer 2100 system. Clustering of the index-coded samples was performed on a cBot Cluster Generation System using a HiSeq PE Cluster Kit v4-cBot-HS (Illumia, San Diego, CA, USA) according to the manufacturer’s instructions. The cDNA libraries were sequenced on an Illumina Hiseq platform and 150-bp paired-end reads were generated.

### 2.3. Gene Annotation and Mapping

Clean reads were derived from raw reads by removing adapter reads, low-quality reads, and reads containing poly-N and ambiguous bases using the trimmomatic [28]. Clean reads were aligned to the reference genome [29] using Hisat2 [30] for gene expression analysis. Differential genes expression (DGE) in ds*NlFoxO* versus ds*Gfp* was analyzed using DEseq2 [31]. False discovery rate (FDR) was used to measure the threshold *p*-value in multiple tests, and transcripts with fold change ≥2 and FDR < 0.05 were subjected to differentially expressed gene (DEG) analysis [32,33].

### 2.4. Gene Ontology and Kyoto Encyclopedia of Genes and Genomes Pathway Analyses

Gene ontology (GO) and Kyoto Encyclopedia of Genes and Genomes (KEGG) pathways were analyzed using an online platform (https://www.omicshare.com/tools/) on 15 February 2021. GO terms with corrected *p*-value <0.05 were defined as significantly enriched GO terms [29]. DEGs were used for KEGG analysis to identify markedly enriched metabolic pathways or signal transduction pathways with corrected *p*-value < 0.05 [34].

## 3. Results

### 3.1. Overview of Transcriptomes

A total of 36 cDNA libraries were prepared from 12 samples with three biological replicates each, then used for Illumina sequencing. More than 44.6 million raw reads were generated from each cDNA library with Q30 values in all libraries exceeding 93.85%. Following the removal of adaptors, poly-N and low-quality reads, more than 43.4 million clean reads were retained for each sample (Table S1). The mapping rate of clean reads against the *N. lugens* reference genome ranged from 66.25% to 74.78%.

### 3.2. DEGs in Wing Buds of 5th-Instar Nymphs Treated with dsNlFoxO and dsGfp 

**Figure 1:**
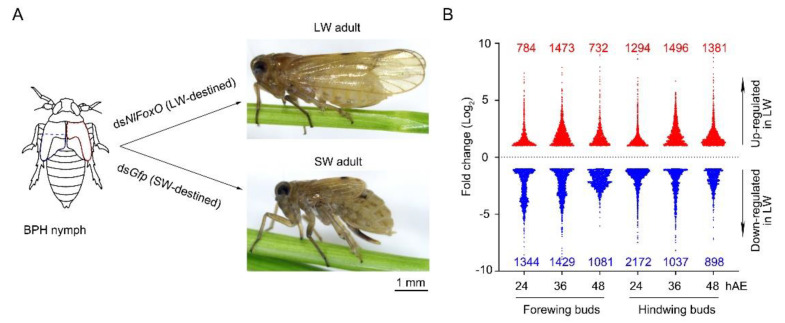
Schematic depiction of long-winged (LW)- and short-winged (SW)- destined BPHs, and differentially expressed genes (DEGs) in wing buds. (**A**) 4th-instar nymphs were microinjected with ds*NlFoxO* and ds*Gfp* to generate LW and SW destined BPHs, respectively. Forewing and hindwing buds were dissected from 5th-instar nymphs at 24, 36, and 48 h after ecdysis (hAE), then collected for RNA-sequencing. (**B**) DEGs in LW-destined (ds*NlFoxO*) versus SW-destined (ds*Gfp*) wing buds.

Using the fold change ≥2 and FDR < 0.05 as criteria, we identified 2128 (784 up-regulated and 1344 down-regulated), 2902 (1473 up-regulated and 1429 down-regulated), and 1813 (732 up-regulated and 1081 down-regulated) DEGs in forewing buds of ds*NlFoxO*-treated 5th-instar nymphs at 24, 36, and 48 hAE, respectively, relative to ds*Gfp* treatment. For hindwing buds, 3466 (1294 up-regulated and 2172 down-regulated), 2533 (1496 up-regulated and 1037 down-regulated), and 2279 (1381 up-regulated and 898 down-regulated) DEGs were identified in ds*NlFoxO*-treated 5th-instar nymphs at 24, 36, and 48 hAE, respectively (Figure 1B).

### 3.3. Functional Enrichment Analysis of DEGs in Forewing and Hindwing Buds at 24 hAE

**Figure 2:**
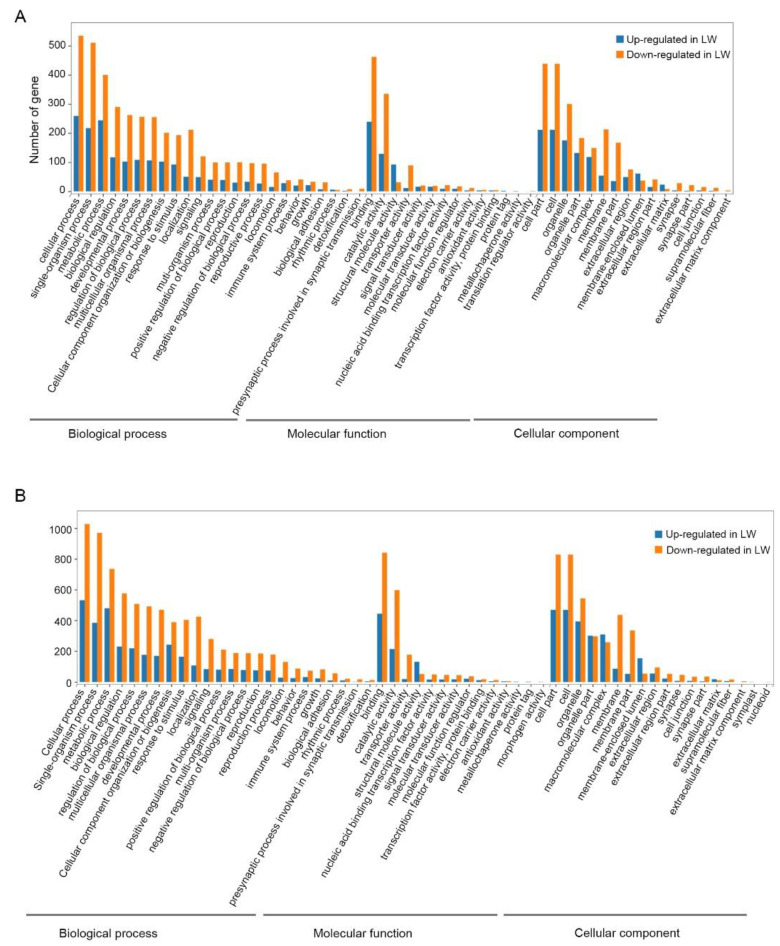
Gene Ontology (GO) classification of differentially expressed genes in forewing (**A**) and hindwing (**B**) buds of 24 hAE-5th-instar nymphs with *NlFoxO* and *Gfp* knockdown. GO analysis is summarized as three main categories: cellular component (CC), molecular function (MF), and biological process (BP).

**Figure 3:**
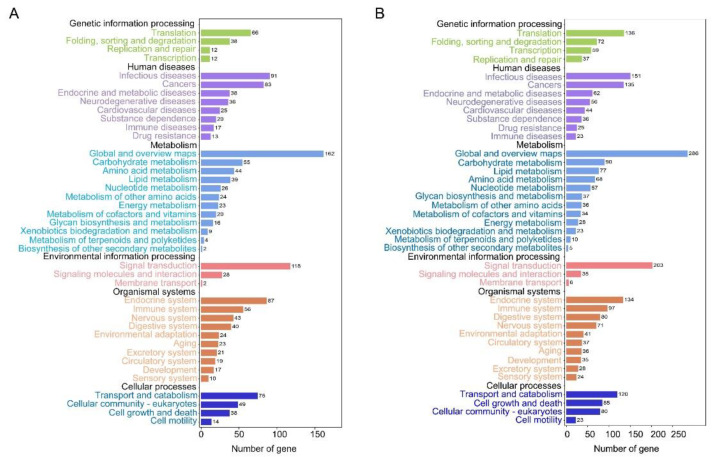
Kyoto Encyclopedia of Genes and Genomes (KEGG) pathway classification of DEGs in the forewing (**A**) and hindwing (**B**) buds of 24 hAE-5th-star nymphs with *NlFoxO* and *Gfp* knockdown.

To better understand the regulatory mechanisms of long wing development regulated by ds*NlFoxO*, DEGs were used for GO term and KEGG pathway enrichment analyses. For forewing buds at 24 hAE, 2128 DEGs were enriched in 54 GO terms associated with 24 biological process (BP) categories (GO:0008150), 16 cellular component (CC) categories (GO:0005575), and 14 molecular function (MF) categories (GO:0003674) with FDR < 0.05 as the criterion (Table S2; Figure 2A). The top-ranked terms included cellular process (259 up-regulated and 534 down-regulated genes), binding (239 up-regulated and 462 down-regulated genes), and cell part (211 up-regulated and 438 down-regulated genes; Figure 2A). KEGG enrichment pathway analysis showed that the 2128 DEGs of forewing buds at 24 hAE were annotated into six KEGG classes (Figure 3A); genetic information processing (four pathways), metabolism (12 pathways), human diseases (eight pathways), environmental information processing (three pathways), organismal systems (10 pathways), and cellular processes (four pathways) (Figure 3A). The “global and overview maps” pathway was the most enriched, followed by “signal transductions” and “infectious diseases” pathways. 

**Figure 4:**
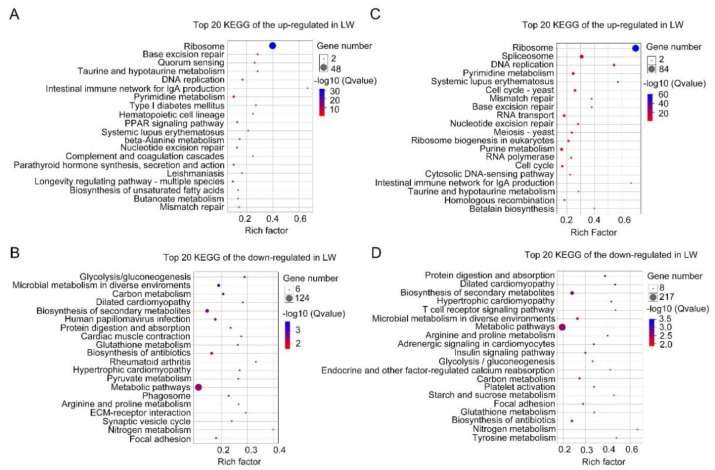
Top 20 enriched KEGG pathways of DEGs in 24 hAE-5th-instar nymphs with *NlFoxO* and *Gfp* knockdown. (**A**,**B**) Top 20 enriched KEGG pathways of up-regulated and down-regulated genes in forewing buds, respectively. (**C**,**D**) Top 20 enriched KEGG pathways of up-regulated and down-regulated genes in hindwing buds, respectively. The *q*-values in different colors denote significant enrichment. Different sizes of dots represent the number of genes enriched in each pathway.

Subsequently, up-regulated and down-regulated DEGs were further separately collated for KEGG pathway analysis. Out of 784 up-regulated DEGs, only 54 genes were assigned to KEGG pathways (*p*-values < 0.05; Table S3; Figure 4A), and the ribosome pathway (ko03010) was at the top of the list (*q*-values < 0.05; Figure 4A). Out of 1344 down-regulated DEGs, 124 out of 1344 genes were the most significantly enriched in the metabolic pathway (ko01100; Table S3; Figure 4B).

For hindwing buds at 24 hAE, 3466 DEGs including 1294 up-regulated and 2172 down-regulated genes were assigned to 54 GO terms (Table S4; Figure 2B), among which the terms cellular process (531 up-regulated and 1027 down-regulated genes), cell part (468 up-regulated and 828 down-regulated genes), and binding (443 up-regulated and 841 down-regulated genes) contained the most enriched DEGs (Figure 2B). This observation is in line with GO analysis of forewing buds at 24 hAE. The 3466 DEGs were classified into six KEGG classes, and the top-ranked pathways included “global and overview maps” and “signal transduction” (Figure 3B). For up-regulated DEGs in hindwings, 218 out of 1294 genes could be matched to KEGG pathways. Analogous to forewings, “ribosome” was the most enriched term, followed by spliceosome, and DNA replication (ko03030; Table S3; Figure 4C). For down-regulated DEGs, 217 out of 1081 genes were assigned to KEGG pathways, and “metabolic” was the most significantly enriched pathway (Table S3; Figure 4D), consistent with forewing buds at 24 hAE.

### 3.4. Functional Enrichment Analysis of DEGs in Forewing and Hindwing Buds at 36 hAE

**Figure 5:**
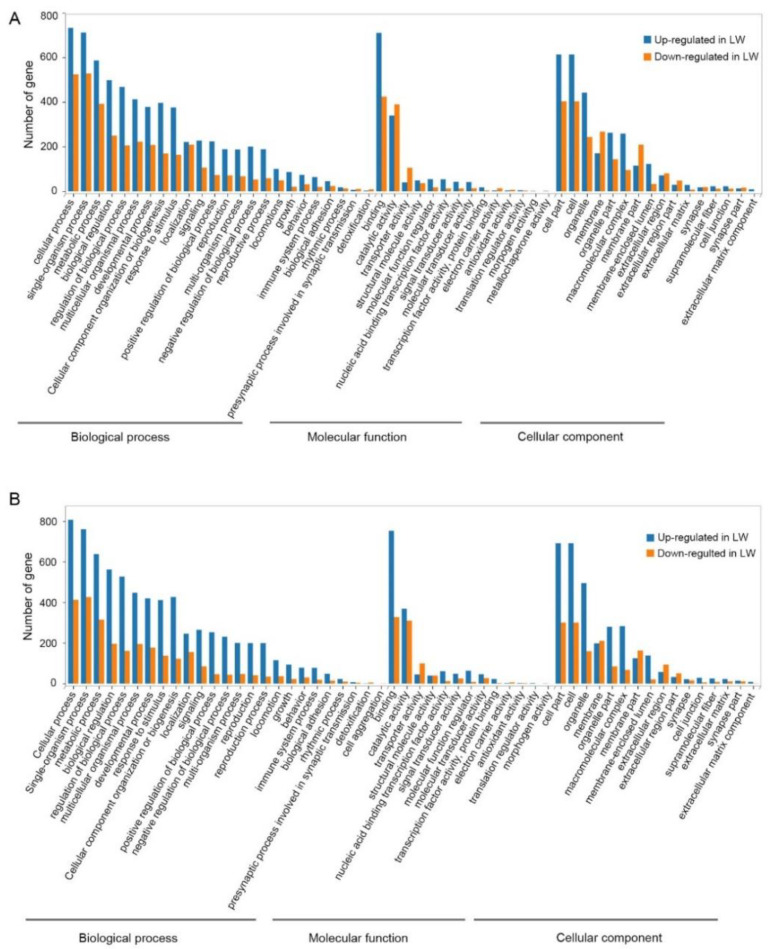
GO classification of DEGs in forewing (**A**) and hindwing (**B**) buds of 36 hAE-5th-instar nymphs with *NlFoxO* and *Gfp* knockdown. GO is summarized as three main categories (CC, MF and BP).

**Figure 6:**
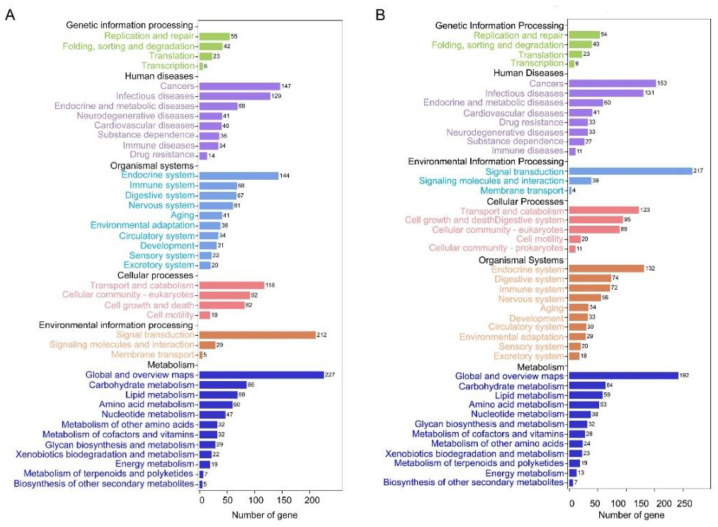
KEGG pathway classification of DEGs in the forewing (**A**) and hindwing (**B**) buds of 36 hAE-5th-star nymphs with *NlFoxO* and *Gfp* knockdown.

**Figure 7:**
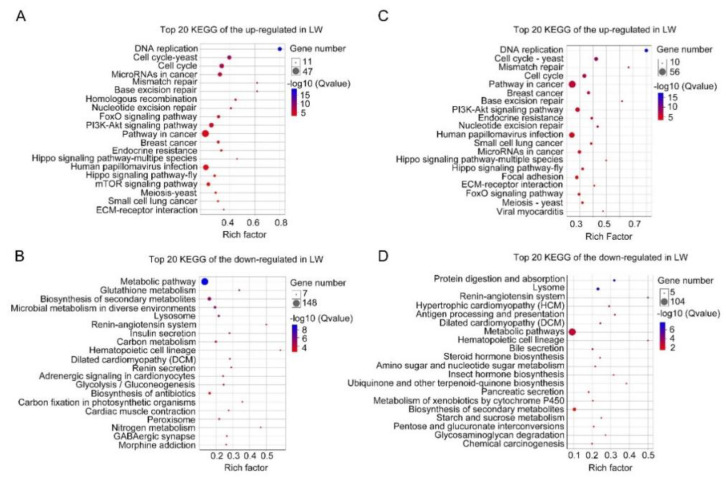
Top 20 enriched KEGG pathways of DEGs in 36 hAE-5th-instar nymphs with *NlFoxO* and *Gfp* knockdown. (**A**,**B**) Top 20 enriched KEGG pathways of up-regulated and down-regulated genes in forewing buds, respectively. (**C**,**D**) Top 20 enriched KEGG pathways of up-regulated and down-regulated genes in hindwing buds, respectively. The *q*-values in different colors denote significant enrichment. Different sizes of dots represent the number of genes enriched in each pathway.

For forewing buds at 36 hAE, 2902 DEGs (1473 up-regulated and 1429 down-regulated) were categorized into 24 BP, 14 MF and 16 CC GO terms based on FDR < 0.05 (Table S5). As shown in Figure 5, cellular process (732 up-regulated and 524 down-regulated genes), binding (710 up-regulated and 424 down-regulated genes), and cell part (613 up-regulated and 403 down-regulated genes) were the top-ranked GO terms, respectively. KEGG analysis showed that DEGs in forewing buds at 36 hAE were significantly enriched in six KEGG classes including genetic information processing (4 pathways), human diseases (eight pathways), organismal systems (10 pathways), cellular processes (four pathways), environmental information processing (three pathways), and metabolism (12 pathways) (Figure 6A). The “global and overview maps” and “signal transductions” were the two most enriched KEGG pathways (Figure 6A), including 227 and 212 genes, respectively. KEGG analysis of the 1473 up-regulated genes showed that the top 20 enriched pathways included DNA replication (ko03030), cell-cycle yeast (ko04111), and cell-cycle (ko04110), suggesting that these pathways played important roles in long wing development (Figure 7A). KEGG analysis of the 1429 down-regulated genes showed that the metabolic pathway was apparently the most enriched pathway (Figure 7B).

For hindwing buds at 36 hAE, 2533 (1496 up-regulated and 1037 down-regulated) DEGs were annotated using GO classifications, including 25 BP, 13 MF, and 16 CC terms, among which cellular process (807 up-regulated and 412 down-regulated), binding (753 up-regulated and 327 down-regulated), and cell part (691 up-regulated and 300 down-regulated) were the top-ranked terms (Table S6; Figure 5B). In line with forewing buds at 36 hAE, the 2533 DEGs in hindwing buds at 36 hAE were distributed in 41 KEGG pathways, and the “global and overview maps” and “signal transductions” pathways contained the most DEGs (Figure 6B). KEGG analysis of the 1496 up-regulated genes showed that DNA replication and cell cycle were significantly enriched (Figure 7C), consistent with the results for forewing buds at 36 hAE. For the 1037 down-regulated DEGs, a metabolic pathway contained the most DEGs in hindwing buds at 36 hAE (Figure 7D).

### 3.5. Functional Enrichment Analysis of DEGs in Forewing Buds and Hindwing Buds at 48 hAE

**Figure 8:**
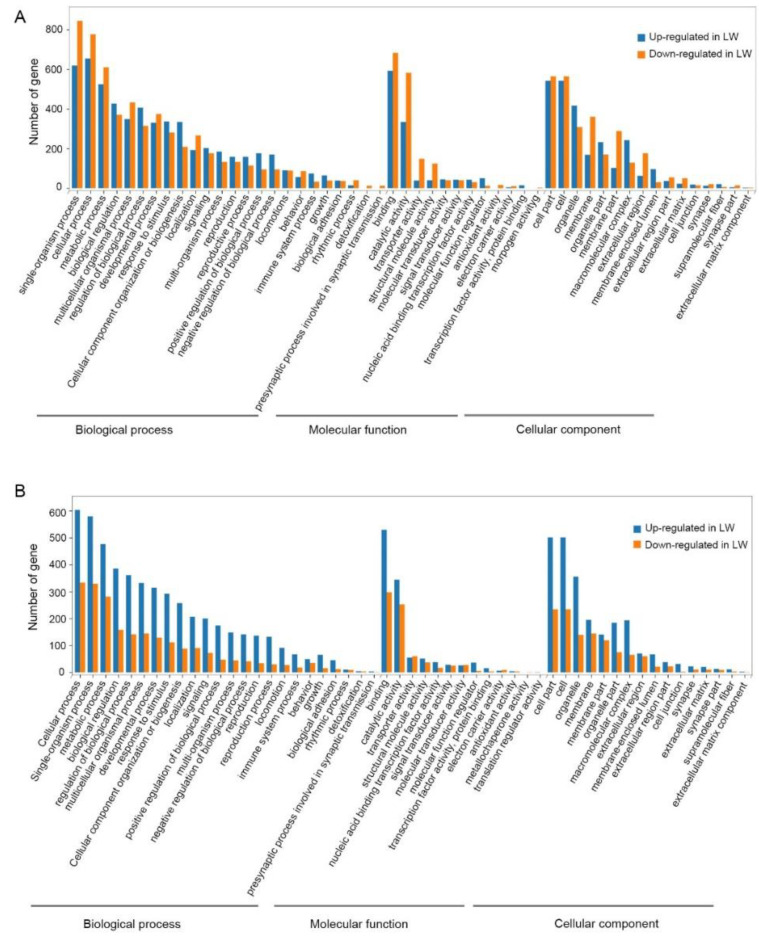
GO classification of DEGs in forewing (**A**) and hindwing (**B**) buds of 48 hAE-5th-instar nymphs with *NlFoxO* and *Gfp* knockdown. GO was summarized as three main categories (CC, MF and BP).

**Figure 9:**
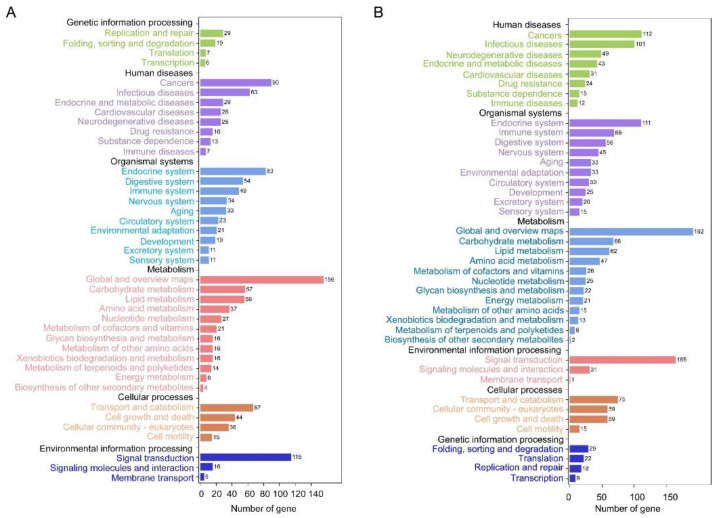
KEGG pathway classification of DEGs in the forewing (**A**) and hindwing (**B**) buds of 48 hAE-5th-star nymphs with *NlFoxO* and *Gfp* knockdown.

**Figure 10:**
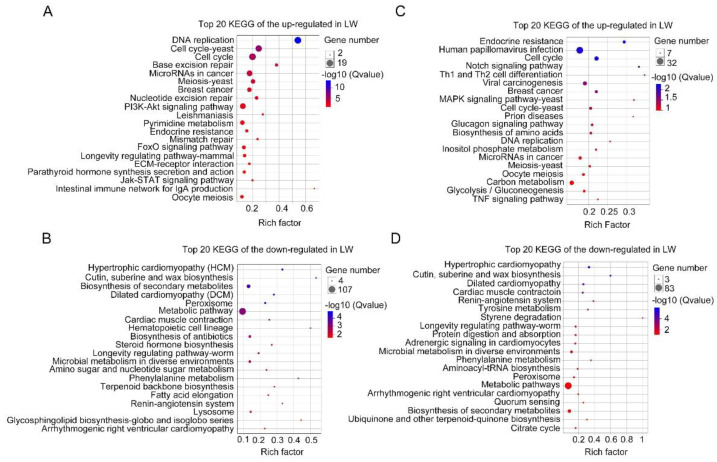
Top 20 enriched KEGG pathways of DEGs in 48 hAE-5th-instar nymphs with *NlFoxO* and *Gfp* knockdown. (**A**,**B**) Top 20 enriched KEGG pathways of up-regulated and down-regulated genes in forewing buds, respectively. (**C**,**D**) Top 20 enriched KEGG pathways of up-regulated and down-regulated genes in hindwing buds, respectively. The *q*-values in different colors denote significant enrichment. Different sizes of dots represent the number of genes enriched in each pathway.

For forewing buds at 48 hAE, the 1813 (732 up-regulated and 1081 down-regulated) DEGs were mapped to 52 GO terms (Table S7; Figure 8A). Analogous to forewing buds at 24- and 36-hAE, most genes were assigned to single-organism processes, binding, and cell part terms (Figure 8A). KEGG analysis showed that “global and overview maps” and “signal transductions” were the two most enriched pathways (Figure 9A), including 156 and 115 genes, respectively. In addition, KEGG analysis of the up-regulated genes showed that the top enriched pathways included DNA replication and cell cycle (Table S3; Figure 10A), while metabolic pathways was the most significantly enriched pathway in the down-regulated genes (Table S3; Figure 10B). 

For hindwing buds at 48 hAE, greater percentages of the 2279 DEGs (1381 up-regulated and 898 down-regulated) were mapped to the single-organism process, binding, and cell part terms (Table S7; Figure 8B). KEGG analysis showed that “global and overview maps” and “signal transductions” were the two most enriched pathways (Figure 9B), including 192 and 165 genes, respectively. The top enriched pathways in the up-regulated genes included endocrine resistance, human papillomavirus infection, and cell cycle (Table S3; Figure 10C), while “metabolic pathways” was the most significantly enriched pathway in the down-regulated genes (Table S3; Figure 10B). 

### 3.6. Expression Pattern of Cell Proliferation-Associated Genes in LW- and SW-Destined Wing Buds

**Figure 11:**
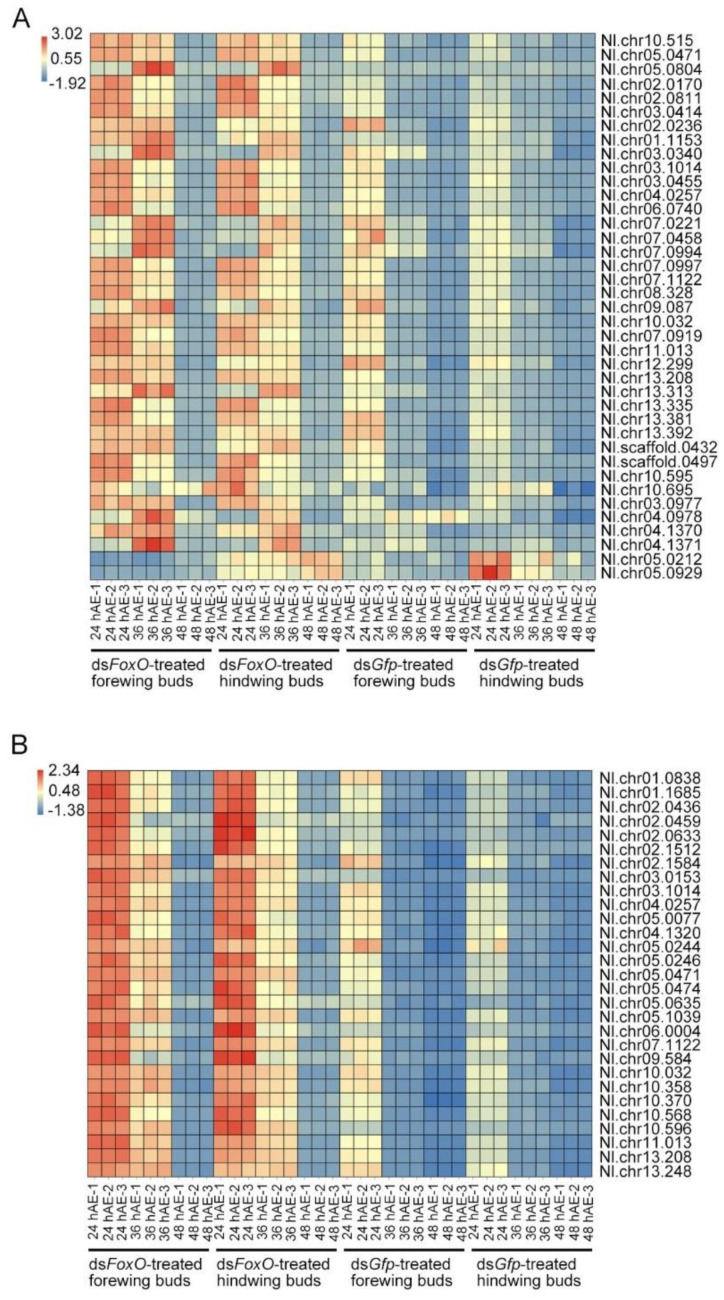
Heatmap of DEGs in cell cycle and DNA replication pathways. Transcripts with fold change ≥ 2 and FDR < 0.05 were subjected to heatmap analysis.

Because the cell cycle and DNA replication pathways were repeatedly detected in up-regulated genes of LW-destined buds (ds*NlFoxO* treatment; Figure 4A,C, Figure 7A,C and Figure 10A,C), we investigated their expressional profile across 5th-instar stage using heat-maps with *p*-value <0.05 as a cutoff criterion. A total of 39 cell-cycle- and 25 DNA-replication-associated genes were significantly regulated by ds*NlFoxO* versus ds*Gfp* in forewing and hindwing buds (Table S9; Figure 11). For both forewing and hindwing buds treated with ds*NlFoxO*, most genes exhibited a higher expression at 24-, and 36-hAE relative to 48 hAE (Figure 11), indicating that cells of LW-destined wings might actively proliferate within the first 36 h of 5th-instar nymphs. In contrast, for both forewing and hindwing buds treated with ds*Gfp*, a higher expression level was detected at 24 hAE than 36- and 48-hAE, indicating that wing cells in SW-destined wings might proliferate within the first 24 h of 5th-instar nymphs. Thus, these observations indicated that the duration of cell proliferation might contribute significantly to long wing development in ds*NlFoxO*-treated BPHs.

## 4. Discussion

Previous studies showed that inactivation of *NlFoxO* resulted in a switch from SW to LW morphs in BPH [4,5,35]. To further explore the molecular basis regulated by *Nl*FoxO, transcriptomic profiles were conducted on LW- and SW-destined wing buds of 5th-instar nymphs, the wing-morph decisive stage of BPH [7]. We showed that knockdown of *NlFoxO* significantly altered the expression of 2128, 2902, and 1813 genes in forewing buds of 5th-instar nymphs at 24, 36, 48 hAE, respectively, and simultaneously altered the expression of 3466, 2533, and 2279 genes in hindwing buds. This observation indicated that differentiation of wing morph caused a remarkable change in gene expression in wing cells. 

Based on the comparative transcriptomic data, we noticed that ds*NlFoxO*-treated forewing had a similar gene expression pattern to that of hindwing buds at 24, 36, and 48 hAE. GO analysis showed that DEGs were mostly enriched in cellular process, binding, and cell part terms in forewing and hindwing buds at each time-point (Figure 2, Figure 5 and Figure 8). KEGG pathway analysis showed that the up-regulated DEGs were largely assigned to the ribosome pathway in both forewing and hindwing buds at 24 hAE. However, the top enriched KEGG pathway was switched from ribosome to DNA replication and cell cycle as developmental time proceeded into 36 hAE. This observation suggested that knockdown of *NlFoxO* might increase the expression levels and duration of cell proliferation-associated genes in wing buds, which might be the main driver of long wing development. This assumption was also supported by heatmap analysis on cell-cycle- and DNA-replication-associated genes across the 5th-instar developmental stage (Figure 11). We noticed that genes were highly expressed in ds*NlFoxO*-treated forewing and hindwing at both 24 and 36 hAE. In contrast, cell proliferation-associated genes were only active at 24 hAE in ds*Gfp*-treated forewing and hindwing buds (Figure 11). 

Previous studies in mammals showed that FoxO transcription factors play a major role in cell cycle arrest at the G1/S and G2/M transitions, two checkpoints that are critical in the cellular process [13]. FoxOs promote cell cycle arrest at the G1/S boundary by both up-regulating cell cycle inhibitors (p21 and p27) [36,37] and by repressing cell cycle activators (cyclin D1 and D2) [21,38]. FoxOs arrest cell progression at the G2/M boundary by regulating the expression of cyclin G2 [39], and trigger DNA repair by modulating the expression of growth arrest and DNA damage-inducible protein 45 (*Gadd45*) [40,41]. This regulatory mechanism may explain the decrease in cell number [22,23,24] and cell size [22,23] in *Drosophila* caused by ectopic expression of dFoxO. In the present, our results show that knockdown of *NlFoxO* significantly enhanced the expression of cyclin D2 (Nl.scaffold.0432; Table S9; Figure 11) in wing cells of BPH, which may partially explain the long wing development in BPH upon *NlFoxO* knockdown.

## 5. Conclusions

This study investigated the transcriptional profile of wing-morph transition from SW (ds*Gfp*) to LW (ds*NlFoxO*) in 5th-instar nymphs, the wing-morph decisive stage. Comparative transcriptome analysis revealed that a large percentage of genes (10–19%) were significantly differentially expressed in both forewing and hindwing buds. GO enrichment analysis showed that DEGs were mainly related to cellular process, binding, and cell part categories. The up-regulated genes were significantly enriched in DNA replication (ko03030), and cell cycle (ko04110 and ko04111) pathways in KEGG analysis. Thus, the duration of cell proliferation might contribute significantly to long wing development modulated by *NlFoxO*.

## Data Availability

The clean data from the RNA-seq were submitted to GenBank (BioProject accession number: PRJNA715841).

## Supplementary Materials

The following are available online at https://www.mdpi.com/article/10.3390/insects12050413/s1. Supplementary Table S1: Data quality of RNA-seq. Supplementary Table S2: Differentially expressed genes of GO classification in forewing at 24 hAE. Supplementary Table S3: KEGG enrichment of differentially expressed genes in forewing and hindwing buds at 24, 36, and 48 hAE. Supplementary Table S4: Differentially expressed genes of GO classification in hindwing at 24 hAE. Supplementary Table S5: Differentially expressed genes of GO classification in forewing at 36 hAE. Supplementary Table S6: Differentially expressed genes of GO classification in hindwing at 36 hAE. Supplementary Table S7: Differentially expressed genes of GO classification in forewing at 48 hAE. Supplementary Table S8: Differentially expressed genes of GO classification in hindwing at 48 hAE. Supplementary Table S9: Differentially expressed genes associated with cell cycle and DNA replication.
